# K_Ca_3.1-dependent uptake of the cytotoxic DNA-binding dye Hoechst 33258 into cancerous but not healthy cervical cells

**DOI:** 10.1074/jbc.RA120.013997

**Published:** 2020-11-23

**Authors:** Maurish Bukhari, Han Deng, Darren Sipes, Marisa Ruane-Foster, Kayla Purdy, Craig D. Woodworth, Shantanu Sur, Damien S.K. Samways

**Affiliations:** Department of Biology, Clarkson University, Potsdam, New York, USA

**Keywords:** ATP, Ca^2+^, cancer, drug delivery, Hoechst, ion channel, K_Ca_3.1, permeability, plasma membrane, ATP, adenosine 5’triphosphate, CXT, cervical cancer cell line, DiSBAC2(3), bis-(1,3-diethylthiobarbituric acid)trimethine oxonol, ECTO, primary ectocervical epithelial cell line, H33258, Hoechst 33258, K_Ca_3.1, intermediate conductance Ca^2+^-activated K^+^ channel, TZ, primary transformation zone epithelial cell line

## Abstract

The poor and nonselective penetration of current chemotherapeutics across the plasma membranes of cancer cells, which is necessary for the targeted disruption of the intracellular machinery, remains a major pharmaceutical challenge. In several cell types, including mast cells and macrophages, exposure to extracellular ATP is known to stimulate passive entry of large and otherwise membrane impermeable cationic dyes, which is usually attributed to conduction through ionotropic P2X receptors. Here, we report that elevations in cytosolic Ca^2+^ stimulate the rapid uptake and nuclear accumulation of a DNA-binding fluorescent cation, Hoechst 33258 (H33258), in cervical cancer cells. The H33258 uptake was dependent on activation of intermediate conductance Ca^2+^-activated K^+^ channels (K_Ca_3.1), and direct stimulation of the channel with the activators SKA 31 and DCEBIO was sufficient to induce cellular uptake of H33258 directly. In contrast to the results from cancerous cervical cells, K_Ca_3.1-dependent H33258 uptake was rarely observed in epithelial cells derived from the ectocervix and transformation zone of healthy cervical tissue. Furthermore, whole-cell patch clamp experiments and assessment of membrane potential using the slow voltage-sensitive dye bis-(1,3-diethylthiobarbituric acid)trimethine oxonol revealed a significant difference in functional K_Ca_3.1 activity between cancerous and healthy cervical epithelial cells, which correlated strongly with the incidence of K_Ca_3.1-dependent H33258 uptake. Finally, we show that activation of K_Ca_3.1 channels caused a modest but significant sensitization of cancer cells to the growth suppressant effects of H33258, lending plausibility to the idea of using K_Ca_3.1 channel activators to enhance cell penetration of small cationic toxins into cancer cells expressing these channels.

In a previous article, we demonstrated that application of extracellular ATP stimulated the rapid uptake and accumulation of a small molecule DNA-binding cationic dye, Hoechst 33258 (H33258), into cultured cervical cancer cells in a manner dependent on both the ATP and H33258 concentrations (1). Moreover, analysis of a wide spectrum of cervical epithelial cell cultures derived from both normal and cancerous tissue revealed that the uptake was preferentially observed in cancerous cells. DNA-binding dyes such as H33258 commonly exhibit cytotoxic profiles, and this dye was originally synthesized under the name Pibenzimol, which underwent clinical trials for efficacy against pancreatic cancer with poor results (2, 3). We argued that the observed ATP-evoked uptake and accumulation of these dyes might reveal a strategy by which cancer cells could be selectively sensitized to anticancer drugs. Given the substantial evidence for ATP-gated P2XR7 ion channels being capable of conducting bulky cationic DNA-binding dyes, such as YO-PRO1 (4, 5, 6), we were surprised to discover that the mechanism of H33258 permeation was independent of P2XR7. Rather, our evidence indicated that the uptake was dependent on the activation of the ATP-sensitive G protein-coupled P2Y receptors and recruitment of an unidentified downstream transport pathway.

The majority of anticancer chemotherapeutics have intracellular targets and must therefore traverse the plasma membrane to be effective. Nevertheless, many current drugs, including the cisplatin derivatives and doxorubicin, have relatively poor passive membrane permeability. In the case of cisplatin, cell penetration is already known to be in part facilitated by endogenous transport pumps (7) and channel proteins (8). Coming up with new and improved means of facilitating drug entry, specifically into cancer cells, is thus a major goal of current research. With this in mind, we sought to reveal the mechanism by which ATP stimulates the uptake and accumulation of H33258 into cancer cells, with the understanding that this might provide a means of selectively sensitizing these cells to cytotoxic cationic drugs. We show that the activation of intermediate conductance Ca^2+^-activated K^+^ channels (K_Ca_3.1) is a critical step in the mechanism of induced H33258 uptake. K_Ca_3.1 channels have been reported to be expressed, and sometimes upregulated, in several cancer cell lines (9, 10, 11, 12, 13, 14, 15). While activation of these channels in cervical cancer cells in our study caused only a modest increase in H33258 cytotoxicity as assessed in cell proliferation experiments, our results give further credence to the plausibility of exploiting endogenous transport pathways for enhanced drug delivery of intracellularly active cytotoxins.

## Results

### ATP-evoked uptake of H33258 was Ca^2+^ dependent

**Figure 1:**
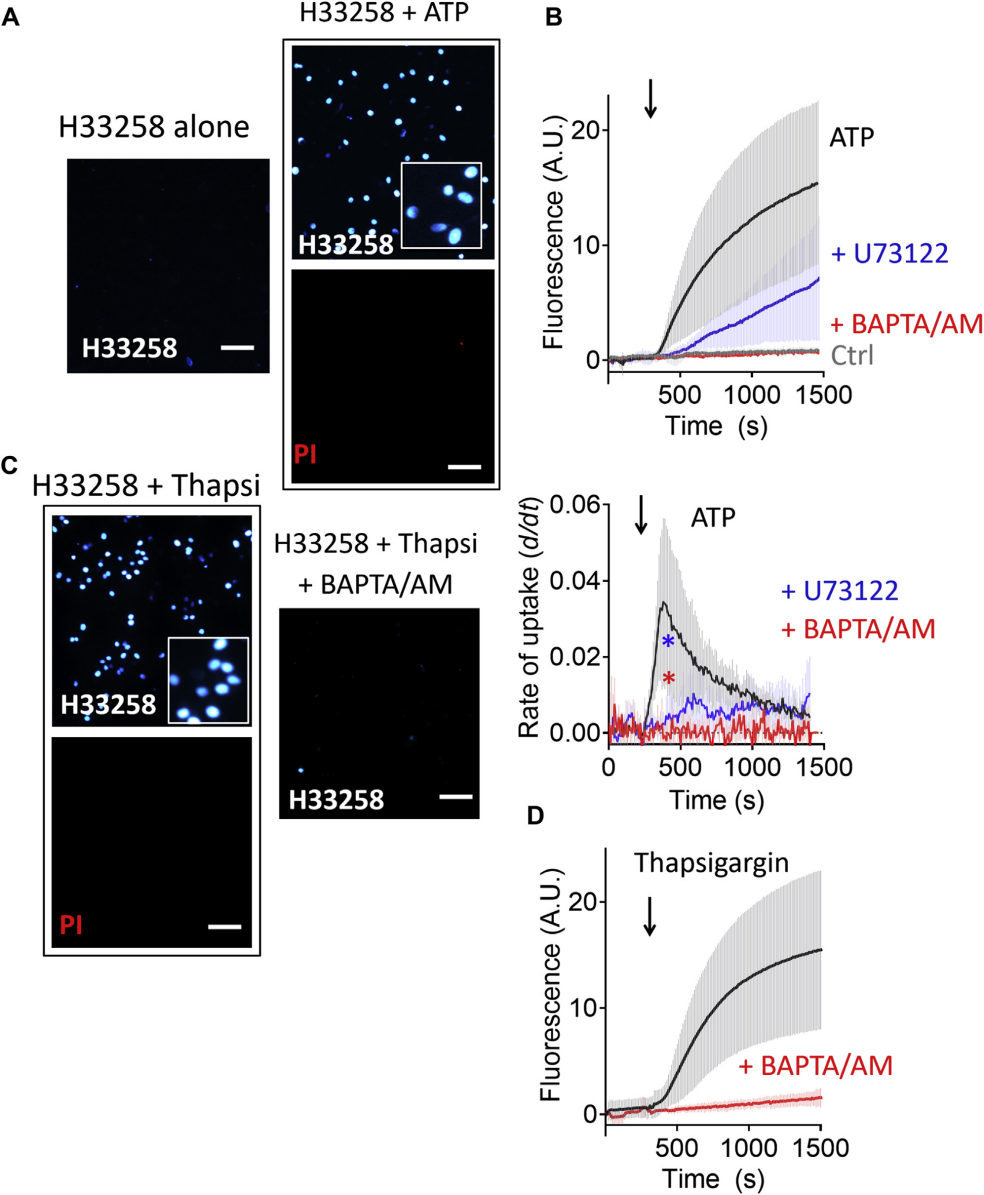
**Cytosolic Ca**^**2+**^**-dependence of ATP-evoked H33258 uptake into cervical cancer cells.***A*, example fluorescence images from CXT2 cervical cancer cell monolayers (×10 magnification) after 20 min of exposure to either H33258 (30 μM) alone (Control) or ATP (100 μM) in the continued presence of H33258 (30 μM). At the end of the experiment, exposure of cells to the vital stain, propidium iodide (PI) (3 μM), for 10 min demonstrated continued viability. *B*, upper panel shows pooled average fluorescence intensity data of H33258 uptake and nuclear accumulation in cells over time (mean ± SD; n = 4–8). The lower panel displays the change in H33258 uptake rate (*d/dt*) over time. Cells exposed to H33258 (30 μM) alone showed negligible uptake over a 20-min period (ctrl). Application of ATP (100 μM) in the continued presence of H33258 caused marked increase in nuclear fluorescence intensity (*black*). ATP-evoked H33258 uptake was significantly suppressed in cells pretreated with the PLC inhibitor, U73122 (1 μM). Also, in cells loaded with the intracellular Ca^2+^ chelator, BAPTA-AM (10 μM), and stimulated with ATP in the absence of added extracellular Ca^2+^, H33258 uptake was significantly suppressed (mean ± SD; n = 3–8). *C*, example fluorescence images of CXT2 cervical cancer cell monolayers after 20 min of exposure to the SERCA inhibitor, thapsigargin (1 μM), in the presence of either H33258 alone or H33258 and BAPTA-AM. *D*, pooled fluorescence data from multiple experiments depicted in (*C*) (mean ± SD; n = 4–7) showing inhibition of thapsigargin-evoked H33258 uptake in cells loaded, (mean ± SD; n = 4–8). The *white scale bars* represent 100 μm (40 μm for digitally magnified insets). BAPTA, 1,2-Bis(2-aminophenoxy)ethane-N,N,N′,N′-tetraacetic acid tetrakis(acetoxymethyl ester).

In a previous study, we demonstrated that extracellular ATP stimulates the uptake and accumulation of H33258 into cervical cancer cells in a concentration-dependent manner that does not require the hydrolysis of ATP (1). We began by reproducing this key finding, using CXT2 cervical cancer cells cultured on glass coverslips mounted into a bath on the stage of a Nikon TE200 inverted microscope and perfused with extracellular solution. 30 μM H33258 was added to the bath at the beginning of the experiments, and fluorescence images were captured every 10 s to monitor H33258 uptake as a function of nuclear fluorescence staining over time. In control experiments, we observed minimal fluorescence over the course of a 20-min period of exposure to H33258 alone, consistent with negligible passive entry of the dye over this time course (Fig. 1, *A*–*B*). In contrast, application of ATP (100 μM) in the continued presence of H33258 resulted in considerable uptake and accumulation of H33258 as evidenced by nuclear staining in a large fraction of cells (Fig. 1, *A*–*B*, black trace). Crucially, cells in which ATP induced H33258 uptake were not co-stained with propidium iodide (10 μM), a bulkier cationic nuclear stain commonly used to assess cell viability. Thus, the H33258 uptake is not merely a result of compromised plasma membrane integrity.

In the previous article, we concluded that the ATP-evoked uptake of H33258 was likely because of activation of P2Y receptors and not the well-known large cation-permeable P2X channels (1). Similar to many keratinocytes, cervical epithelial cells are known to express P2Y2 receptors (16, 17), and we showed that the P2Y2 agonist uridine-5'-triphosphate could stimulate H33258 uptake. We also observed inhibition of ATP-evoked uptake by the selective P2Y1 receptor antagonist, MRS2179, indicating the possible presence of functional P2Y1 receptors also. Both P2Y2 and P2Y1 are conventionally coupled to the G_q_ protein signaling pathway, which activates phospholipase Cβ and results in inositol 1,4,5-triphosphate-dependent mobilization of Ca^2+^ from the endoplasmic reticulum (ER) (18). We hypothesized then that H33258 uptake was a consequence of a transport mechanism stimulated by this signaling pathway. Cervical cancer cells are reported to express G_q_ protein-coupled histamine receptors, which also evoke phospholipase C (PLC)-dependent Ca^2+^ release from the ER (19). Consistent with our hypothesis, stimulation of CXT2 cells with histamine (10 μM) also evoked a modest but measurable uptake of H33258 over a period of 20 min (Fig. S1*A*).

Further consistent with our hypothesis, incubation of CXT2 cells with the PLC inhibitor U73122 (1 μM) significantly suppressed ATP-evoked H33258 uptake (Fig. 1*B*) (*p* = 0.0281). On the other hand, elevating the cytoplasmic Ca^2+^ concentration ([Ca^2+^]_i_) with the SERCA inhibitor thapsigargin (1 μM) strongly stimulated H33258 uptake in the absence of extracellularly applied ATP (Fig. 1, *C*–*D*). In contrast, both ATP- and thapsigargin-evoked H33258 uptake was abolished in cells incubated in 1,2-bis(2-aminophenoxy)ethane-N,N,N′,N′-tetraacetic acid tetrakis(acetoxymethyl ester) (10 μM) for 30 min. Upon intracellular de-esterification, BAPTA strongly buffers cytosolic Ca^2+^ elevations, providing further proof that the evoked H33258 uptake is dependent on an increase in [Ca^2+^]_i_. Stimulation of PLC-dependent Ca^2+^ release from the ER by G_q_ protein-coupled receptors can in turn stimulate store operated Ca^2+^ entry (SOCE) from outside the cell. Under our experimental conditions, ATP-evoked H33258 uptake persisted in the absence of added extracellular Ca^2+^ and in the presence of the broad-spectrum store-operated Ca^2+^ channel blocker, SKF-96365 (Fig. S1).

**Figure 2:**
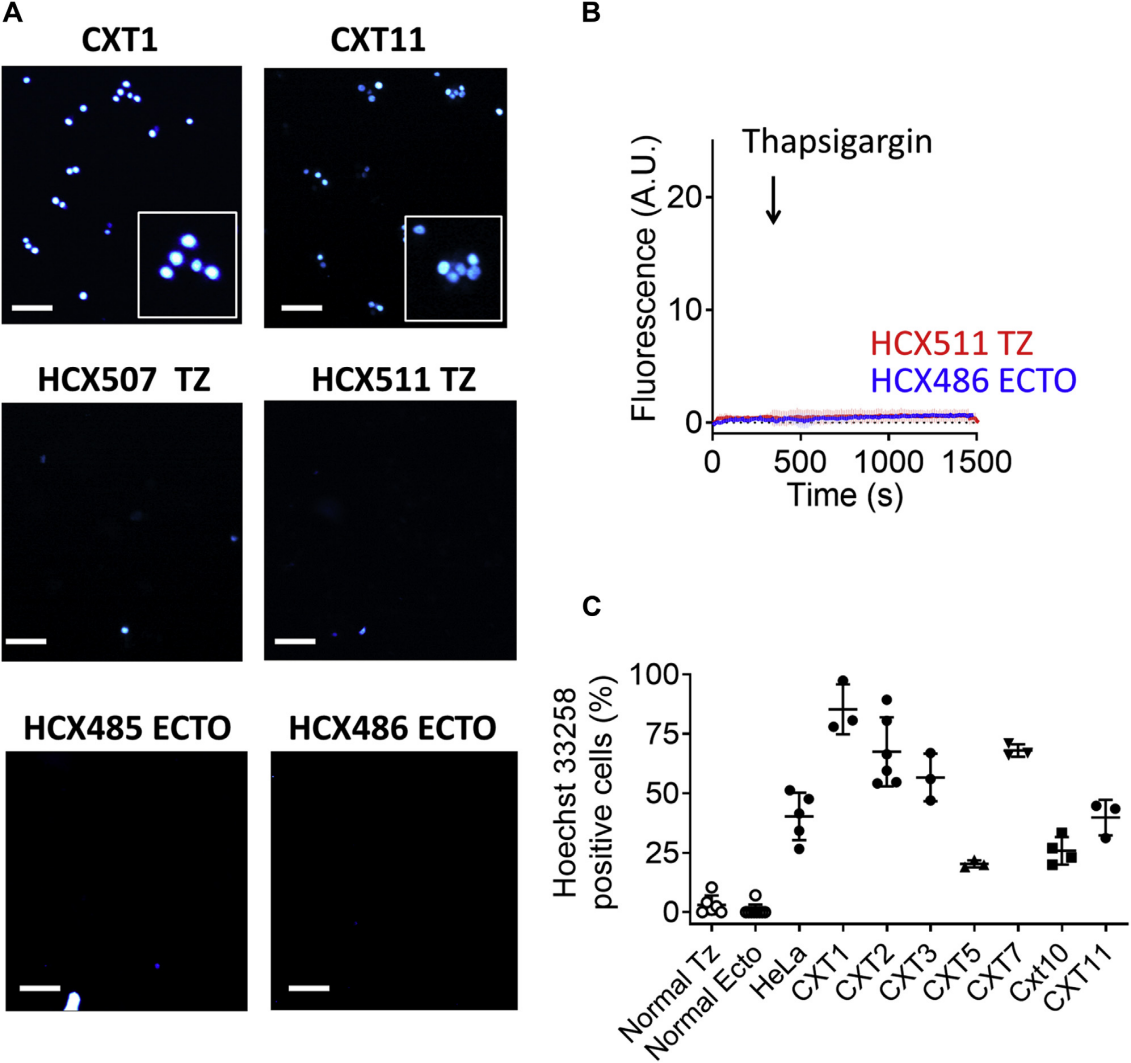
**Thapsigargin-evoked H33258 uptake occurred preferentially in cervical cancer cells relative to normal cervical epithelial cells.***A*, example fluorescence images of cells isolated from cancerous cervix (CXT1 and CXT11) and healthy ectocervix (ECTO) and transformation zone (TZ) tissue (×10 magnification). Images captured after 20 min of exposure to thapsigargin (1 μM) in the continued presence of H33258 (30 μM). *B*, pooled average fluorescence intensity data obtained from two example healthy primary cell lines showing lack of thapsigargin evoked H33258 uptake. *C*, pooled data for the fraction of normal and cancerous cervical cells displaying visible H33258 uptake at x10 magnification after 20 min of exposure to both H33258 (30 μM) and thapsigargin (1 μM). ECTO and TZ cells were obtained from five different patients and pooled together. Each CXT cell line was isolated from tissue obtained from different patients. For comparison, thapsigargin-evoked H33258 uptake experiments were also conducted on the widely known commercial cell line, HeLa. The white scale bars represent 100 μm (40 μm for digitally magnified insets).

Importantly, the ability for thapsigargin to stimulate strong uptake and nuclear accumulation of H33258 was not unique to this particular cervical cancer cell line and was observed to differing degrees in cervical cancer cell lines from tissue originating from eight different patients, including the historical HeLa cell line (Fig. 2, *A*–*C*). This was in keeping with our observations in our previous article that ATP-evoked H33258 uptake was observed in the majority of the cervical cancer cell lines assayed. Also in keeping with our previous results, thapsigargin showed little or no stimulation of H33258 uptake in cervical epithelial cultures derived from healthy cervical tissue obtained from both the transformation zone (TZ) and ectocervix (Fig. 2, *A*–*C*).

### Role of Ca^2+^-sensitive ion channels in mediating H33258 uptake

**Figure 3:**
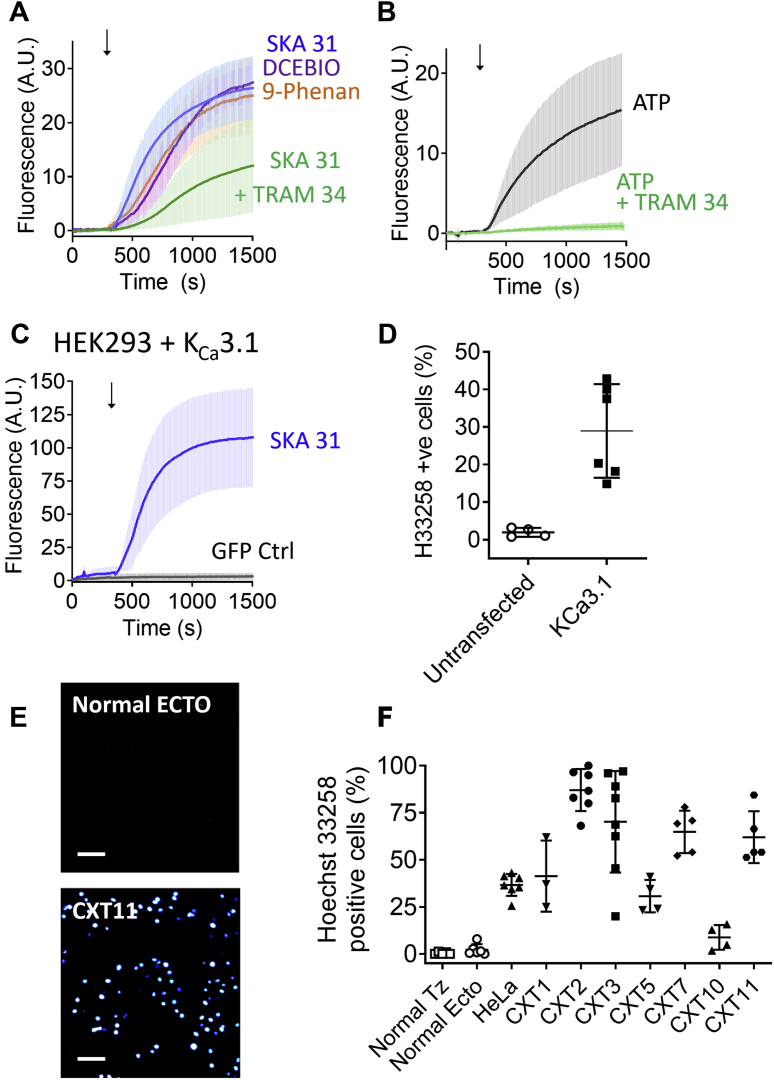
**ATP-evoked H33258 uptake by cervical cancer cells is K**_**Ca**_**3.1-dependent.***A*, exposure of CXT2 cells to the K_Ca_3.1 channel activating agonists, SKA 31 (1 μM), DCEBIO (10 μM), and 9-phenanthrol (10 μM) stimulated substantial H33258 (30 μM) uptake, with the SKA 31-evoked uptake significantly suppressed by the K_Ca_3.1 inhibitor, TRAM 34 (1 μM) (mean ± SD; n = 3–6). *B*, ATP-evoked H33258 uptake was abolished by TRAM 34 (1 μM; mean ± SD; n = 4–6). *C*, HEK293 cells transiently transfected with separate plasmids containing cDNA for human K_Ca_3.1 and GFP responded to SKA 31 (1 μM) exposure with a strong increase in H33258 nuclear fluorescence intensify (mean ± SD; n = 6). In contrast, HEK293 transfected with GFP alone showed negligible SKA 31-evoked H33258 uptake (n = 4). *D*, pooled data for the fraction of HEK 293-GFP and HEK 293-GFP/K_Ca_3.1 cells staining positively for H33258 uptake after exposure to both H33258 (30 μM) for 20 min. *E*, example fluorescence images taken at x10 magnification after 20 min of exposure to H33258 (30 μM) and SKA 31 (1 μM) in cervical cells from heathy ectocervical tissue (ECTO) and CXT11 cervical cancer cells. The *white scale bars* represent 100 μm. *F*, pooled data for the fraction of normal and cancerous cervical cells staining positively for H33258 uptake after 20 min of exposure to both H33258 (30 μM) and SKA 31 (1 μM). TZ, transformation zone.

We predicted that the elevation of Ca^2+^ caused uptake and accumulation of H33258 by stimulating a transmembrane transport pathway. Based on results from our laboratory and others suggesting that several families of ion channels are capable of conducting ionic species of comparable size to H33258 (6, 8, 20), we set out to exclude a dye entry mechanism involving known Ca^2+^-dependent ion channels. We started by looking at cation nonselective ion channels known to be stimulated by cytosolic Ca^2+^, reasoning that these less selective channels were more likely capable of passing H33258 than ion channels with more precise selectivity filters. TRPA1, TRPM4, and TRPM5 channels have previously been shown to be activated by elevated cytosolic Ca^2+^ (21, 22, 23). TRPA1 has also been shown to conduct entry of the bulky DNA-binding dye, YO-PRO1 (24). Nevertheless, neither the presence of the selective TRPA1 inhibitor, A-967079 (1 μM), nor the TRPM5 inhibitor, triphenylphosphine oxide (100 μM), significantly affected ATP-evoked H33258 uptake (Fig. S2*B*). Surprisingly, when we treated cells with the putative antagonist of TRPM4, 9-phenanthrol (10 μM), we observed that this drug alone potently stimulated H33258 uptake (Fig. 3*A*). Further review of the literature revealed that 9-phenanthrol has previously been reported to stimulate K_Ca_3.1 channels (25). Unlike the more weakly cation selective channels that have previously been reported to conduct larger cationic species, K_Ca_3.1 is a member of the highly selective K^+^ channel family and not one we anticipated would likely conduct a relatively large cation like H33258. Nevertheless, we tested the effect of a relatively potent and selective inhibitor of K_Ca_3.1, TRAM 34 (1 μM), on ATP-evoked H33258 uptake and, surprisingly, saw profound suppression (Fig. 3*B*). More compelling still, we found that two other known activators of K_Ca_3.1 channels, SKA 31 (1 μM) and DCEBIO (10 μM), both stimulated robust H33258 uptake upon application to CXT2 cells (Fig. 3*A*). The peak rate of SKA 31-evoked H33258 uptake was strongly and significantly inhibited by TRAM 34, although not abolished (*p* = 0.0002).

Cautious of the selectivity of our activators and inhibitors, we confirmed the critical role of K_Ca_3.1 in the observed H33258 uptake by conducting uptake experiments on mock transfected human embryonic kidney (HEK) 293 cells, and HEK293 cells transiently transfected with plasmid containing the gene for human K_Ca_3.1. Negligible nuclear H33258 uptake in response to SKA 31 (1 μM) was observed in HEK293 cells expressing green fluorescence protein alone, consistent with previous evidence that K_Ca_3.1 is not expressed in the native cell line (26). In contrast, the K_Ca_3.1-expressing HEK293 cells showed a robust SKA 31-evoked H33258 uptake over the course of the experiment (Fig. 3*C*) in a significantly greater number of cells than control (D, *p* = 0.0029). Next, we screened a panel of normal and cancerous cervical cell lines for SKA 31-evoked H33258 uptake, and consistent with our results for ATP and thapsigargin, we observed that the phenomenon was peculiar to cancerous cells (although there was substantial variability in the fraction of responsive cells between lines) (Fig. 3, *E*–*F*).

An interesting observation from our previous article was that ATP-evoked H33258 uptake was also dependent on extracellular Ca^2+^ concentration ([Ca^2+^]_o_). While ATP-evoked marked increased H33258 uptake in extracellular buffer with a similar [Ca^2+^]_o_ to keratinocyte serum-free medium (KSFM) culture media (∼0.1 mM), increasing the [Ca^2+^]_o_ to 2 mM potently suppressed uptake. In the case of SKA 31-evoked uptake, however, we still observed marked nuclear accumulation of H33258 in the presence of 1.8 mM [Ca^2+^]_o_, albeit at a slower rate than in low [Ca^2+^]_o_ extracellular solution (Fig. S2*A*).

Taken together, our data provide compelling evidence that K_Ca_3.1 activation is a prerequisite for Ca^2+^-dependent uptake of H33258 in cervical cancer cells.

### Functional evidence of K_Ca_3.1 upregulation in cervical cancer cells

**Figure 4:**
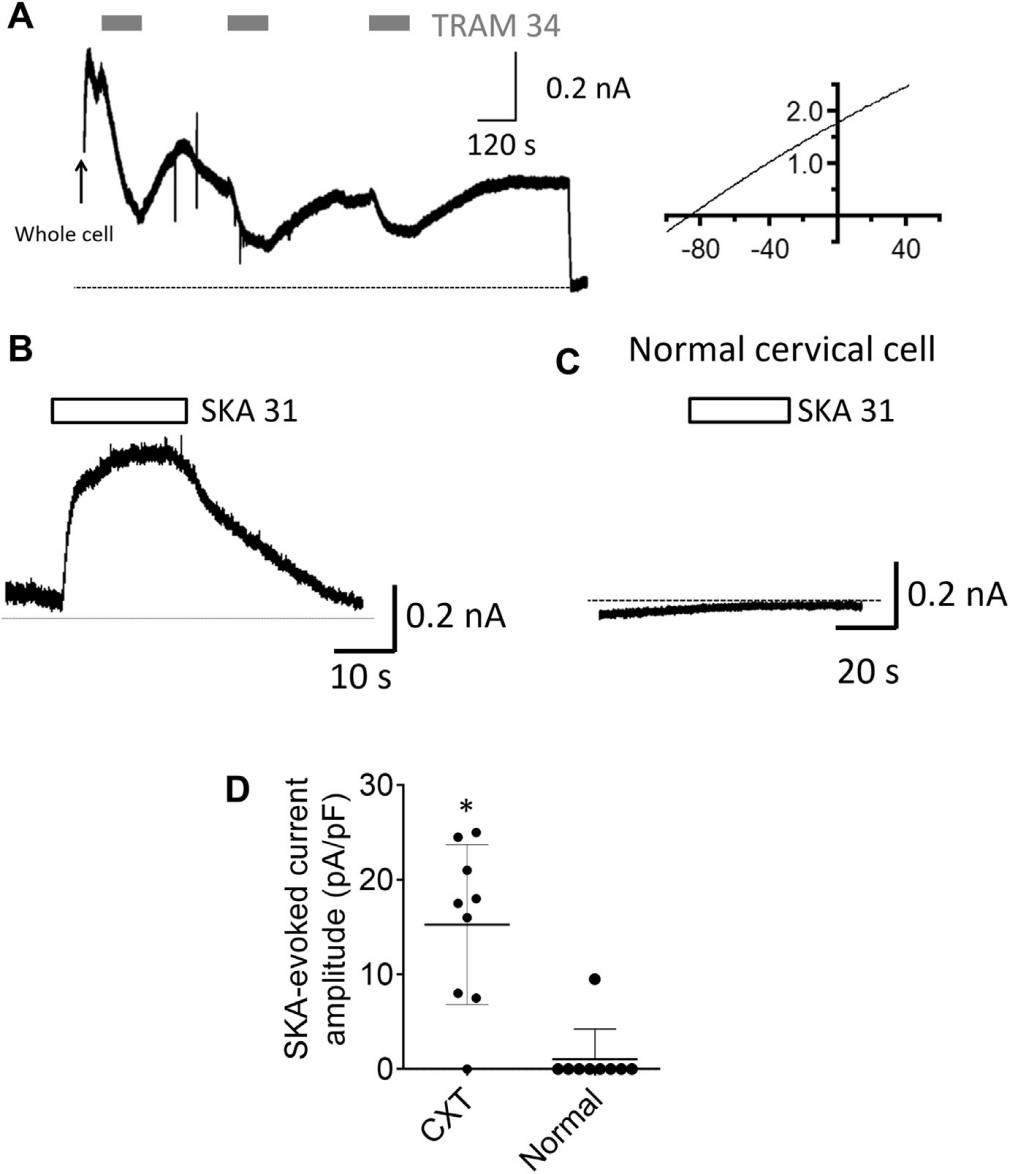
**Electrophysiological evidence for the functional upregulation of K**_**Ca**_**3.1 in cervical cancer cells.** Whole cell broken patch experiments were conducted in single cells voltage clamped at −40 mV. *A*, example current trace obtained from a CXT11 cancer cell patched with a pipette containing a solution with Ca^2+^ buffered to ∼600 nM with EGTA. An outward current was observed immediately upon achieving whole cell configuration. Application of a voltage ramp protocol revealed that current to reverse near the predicted equilibrium potential for K^+^ (E_K_) consistent with an underlying K^+^ conductance (*right inset*). Addition of the K_Ca_3.1 inhibitor, TRAM 34 (1 μM), caused a reversible and reproducible inhibition of the observed outward current. The *dotted line* shows the predicted zero current level upon stepping voltage from −40 mV to the E_K_ value of −90 mV. *B*, example current trace obtained from a CXT2 cancer cell patched with a pipette containing a solution with Ca^2+^ buffered to <100 nM by EGTA. Upon achieving whole cell configuration, a much smaller outward current was observed, which was then significantly increased in amplitude upon application of SKA 31 (1 μM) and declined upon drug washout. *C*, SKA 31 failed to evoke measurable outward currents the majority cervical cells from health ectocervical tissue. *D*, pooled data showing the mean SKA 31-evoked current amplitudes normalized to the individual cell capacitance for cells derived from cancerous (CXT) and healthy cervical tissue. Asterisk denotes statistically significant difference (*p* = 0.0003).

We endeavored to test the reasonable hypothesis that lack of K_Ca_3.1-dependent H33258 uptake in healthy cervical epithelial cells was because of a lower functional expression level of these channels in the plasma membrane compared with cancerous cells. To begin with, we conducted whole cell patch clamp experiments in the voltage clamp configuration to look for evidence of K_Ca_3.1-dependent K^+^ currents in normal and cancerous cervical cells. We used two different methods to stimulate K^+^ currents in these cells. In the first set of experiments, we patched cancerous cervical cells in the whole cell configuration with an intracellular solution in which the Ca^2+^ concentration was buffered to a predicted value of ∼600 nM with EGTA. Most of the K_Ca_ channels are expected to be activated in the presence of ∼600 nM cytosolic Ca^2+^, whether because of direct activation or activation by Ca^2+^/calmodulin as is the case for K_Ca_3.1 (27, 28). Consistent with this, we observed an immediate robust outward current upon breaking into the cell in whole cell configuration (Fig. 4*A*). Assessment of the spontaneous current with a voltage ramp protocol revealed current reversal at a value close to the equilibrium potential for K^+^ predicted by the Goldman Hodgkin Katz voltage equation (29) according to our experimental conditions (E_K_ ∼ −90 mV), indicating a largely K^+^-selective conductance (Fig. 4*A* inset). That this outward current was reversibly inhibited by TRAM 34 (1 μM) is consistent with an underlying Ca^2+^-activated K_Ca_3.1-mediated current. The source of the current remaining in the presence of TRAM 34 is not clear but could be because of other K_Ca_ channel variants or possibly Ca^2+^-activated Cl^−^ channels, which would also elicit an outward current under out experimental conditions.

In the second set of experiments, we patched cells in the whole cell configuration with a pipette solution containing a predicted Ca^2+^ concentration of ∼100 nM. Under the conditions, K_Ca_3.1 activity was anticipated to be low, and indeed upon achieving whole cell status, there was only a very small indication of an outward current (usually little more than a decrease in the leak current toward zero). However, upon perfusing the patched cancerous cells with SKA 31 (1 μM), which is able to activate K_Ca_3.1 in the presence of resting cytosolic Ca^2+^ concentrations (30), we observed a marked outward current that persisted in the presence of the activator in eight out of nine cells patched (Fig. 4*B*). Consistent with our hypothesis that cervical epithelial cells from healthy tissue expressed lower levels of functional K_Ca_3.1, we observed SKA 31-evoked outward currents in significantly smaller fraction of one of nine cells tested (Fig. 4*C*; *p* = 0.0002).

**Figure 5:**
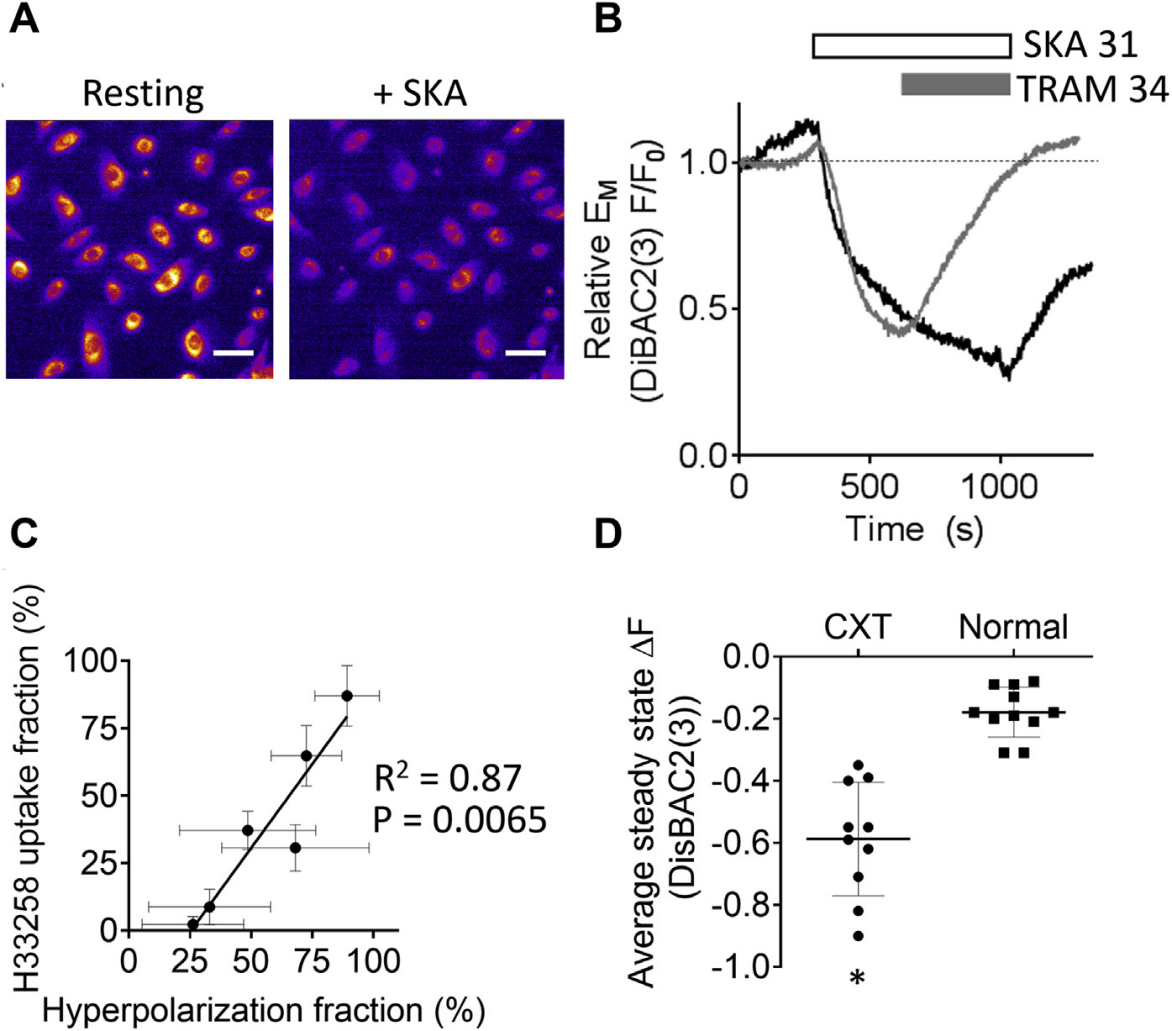
**Effect of SKA 31 on membrane potential in cancerous and healthy cervical epithelial cells as assessed using the voltage-sensitive dye DiSBAC**_**2**_**(3).***A*, representative fluorescence images (×20 magnification) from an experiment in which CXT2 cervical cancer cells bathed in DiSBAC_2_(3) (100 nM) alone (*left panel*) were subsequently stimulated with SKA 31 (1 μM) in the continued presence of DiSBAC_2_(3) (*right panel*). The *white scale bars* represent 50 μm. *B*, representative traces showing the mean DiSBAC_2_(3) fluorescence in CXT2 cells stimulated for 10 min with SKA 31 (1 μM) alone (*black trace*) or SKA 31 followed by TRAM 34 (1 μM) (*gray trace*). *C*, scatter plot showing the correlation between the fraction of cells in which SKA 31 evoked measurable H33258 uptake and the fraction of cells showing measurable SKA-evoked hyperpolarization. Each point represents a distinct cell line, being the mean of amplitude recorded from all experiments conducted in that line. *D*, pooled data showing the mean maximal change in DiSBAC_2_(3) fluorescence upon application of SKA 31 to cancerous CXT cells (each point reports the mean from a single experiment conducted on either CXT2, CXT5 or CXT10 cell lines) and normal (TZ and ECTO cell lines from tissue obtained from at least three patients). Asterisk denotes statistically significant difference (*p* < 0.0001). DiSBAC2(3), bis-(1,3-diethylthiobarbituric acid)trimethine oxonol; ECTO, ectocervix; TZ, transformation zone.

To screen larger numbers of cells for likely functional K_Ca_3.1 channel activity, we used the fluorescence membrane potential–sensitive dye bis-(1,3-diethylthiobarbituric acid)trimethine oxonol (DiSBAC_2_(3)) to monitor changes in membrane potential evoked by K_Ca_3.1 modulators. Under our experimental conditions, the activation of K_Ca_3.1 channels should evoke K^+^ efflux and consequent hyperpolarization. In the presence of DiSBAC_2_(3), which is anionic, this would be observed as a reduction in cytosolic fluorescence as the dye is repelled from the cell by the increasingly negative interior. Cells were incubated in DiSBAC_2_(3) (100 nM) for 20 min before experiments to allow dye equilibration. Consistent with our patch clamp data, we observed SKA 31 (1 μM)-evoked reduction in DiSBAC_2_(3) fluorescence in several cervical cancer cells lines, which was readily reversible by co-application of TRAM 34 (1 μM) (Fig. 5, *A*–*B*). Across at least three independent experiments for each line, we observed SKA31-evoked hyperpolarization in 96 out of 99 CXT2 cells, 30 of 40 CXT5 cells, and 21 of 32 CXT7 cells. In contrast, over the course of 10 separate experiments conducted on normal cervical cell lines obtained from three patients, we observed SKA 31-evoked hyperpolarization in only 45 of 243 cells. Further, in the one cancer cell line that showed only weak SKA 31-evoked H33258 uptake, CXT10, we observed hyperpolarization in only 15 of 135 cells (across six independent experiments). Across both normal and cancerous cell lines, we observed a significant correlation between the fraction of cells exhibiting SKA 31-evoked H33258 uptake and the fraction exhibiting SKA 31-evoked hyperpolarization, consistent with the view that functional K_Ca_3.1 channel expression was essential for the observed stimulated H33258 uptake (Fig. 5*C*). Pooling the data for the amplitudes of the SKA 31-evoked changes in DiSBAC_2_(3) fluorescence, cervical cancer cells expressed a 59 ± 18% (n = 10) mean reduction in DiSBAC_2_(3) fluorescence compared with a significantly smaller 18 ± 8% (n = 11) mean reduction in fluorescence recorded for normal cervical epithelial cells (Fig. 1*D*).

Taken together, our data strongly implicate functional K_Ca_3.1 channels in the selective facilitation of H33258 entry into cervical cancer cells.

### Role of membrane potential in H33258 uptake

**Figure 6:**
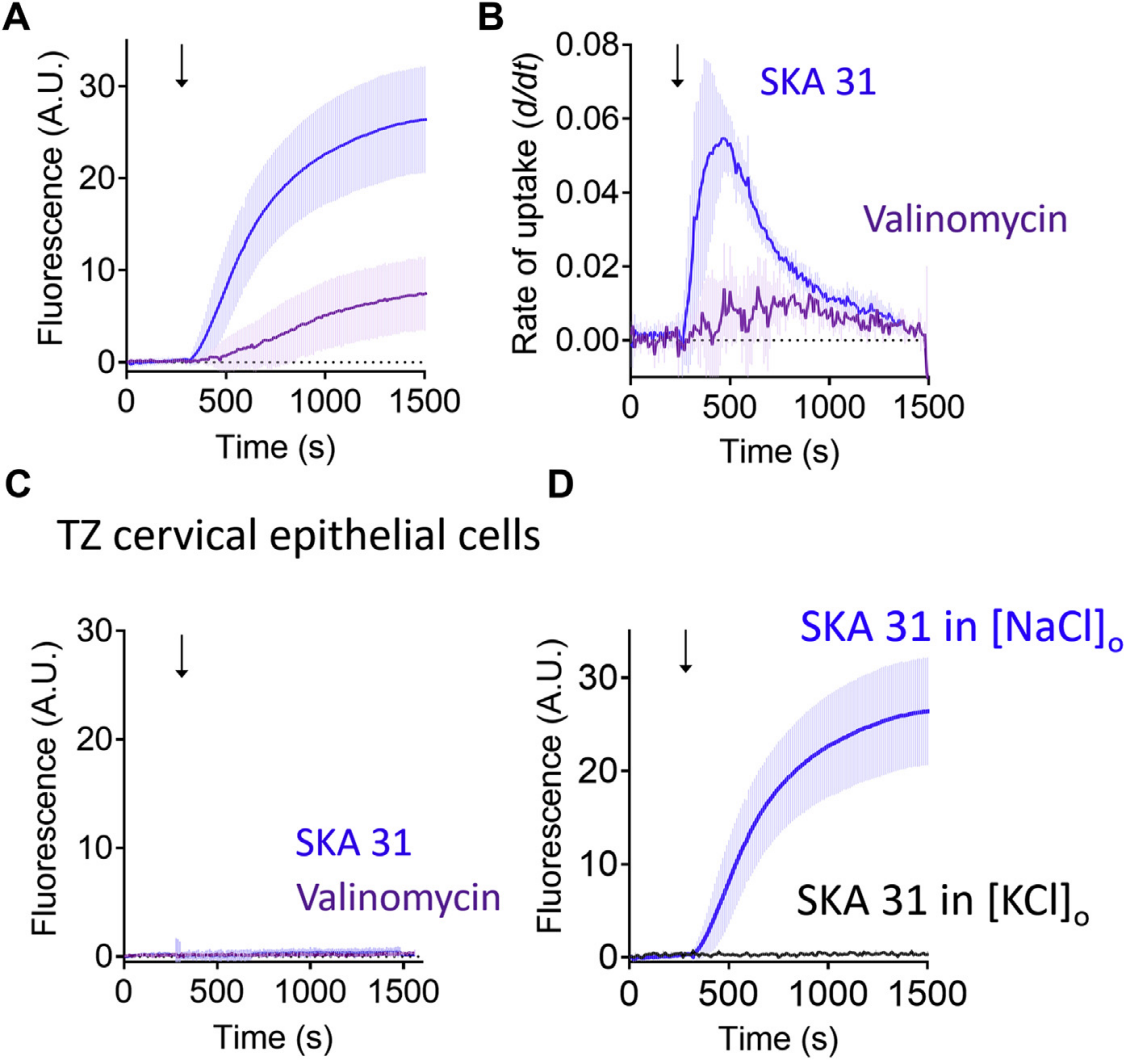
**Effect of changing membrane potential alone on H33258 uptake, in the absence of K**_**Ca**_**3.1 channel activity, in healthy and cancerous cervical cells.***A*, pooled data obtained from CXT2 cells showing modest H33258 uptake in response to the K^+^ ionophore, valinomycin (3 μM; mean ± SD; n = 4). The SKA 31-evoked uptake is shown for comparison. *B*, graph comparing rates of SKA 31 and valinomycin-evoked uptake (produced from data shown in (*A*)). *C*, pooled data showing that neither valinomycin nor SKA 31 evoked measurable H33258 uptake in normal TZ cells (mean ± SD; n = 3). *D*, pooled data showing suppression of SKA 31-evoked H33258 uptake when cell membranes are completely depolarized by substitution of extracellular Na^+^ with equimolar K^+^ (mean ± SD; n = 3–6). TZ, transformation zone.

As we stated in the previous section, the primary consequence of activating K^+^ permeable channels in cells is to cause hyperpolarization. We conducted two sets of experiments to examine whether membrane potential played a role in the observed H33258 uptake. First, we tested if H33258 uptake could be induced by hyperpolarization alone in the absence of K_Ca_3.1 channel activation. The antibiotic valinomycin is a membrane permeable K^+^ carrier ionophore that facilitates the diffusion of K^+^ across the plasma membrane according to its electrochemical gradient, thereby producing hyperpolarization in mammalian cells (31). Valinomycin evoked a measurable but very small uptake of H33258 into CXT2 cells (Fig. 6, *A*–*B*) that was abolished by TRAM 34 (1 μM). Valinomycin did not evoke measurable H33258 uptake in healthy cervical epithelial cells (Fig. 6*C*). This suggests that hyperpolarization alone cannot account for the H33258 uptake caused by activation of K_Ca_3.1 channels.

Next, we tested whether SKA 31 could evoke H33258 uptake under conditions in which the membrane potential was near 0 mV, which was achieved by substituting extracellular NaCl for equimolar KCl (140 mM) (which would clamp E_K_ to approximately zero according to the Goldman Hodgkin Katz model (29)). Under these conditions, SKA 31-evoked H33258 uptake was completely abolished (Fig. 6*D*).

### Dye selectivity of K_Ca_3.1-dependent uptake

**Figure 7:**
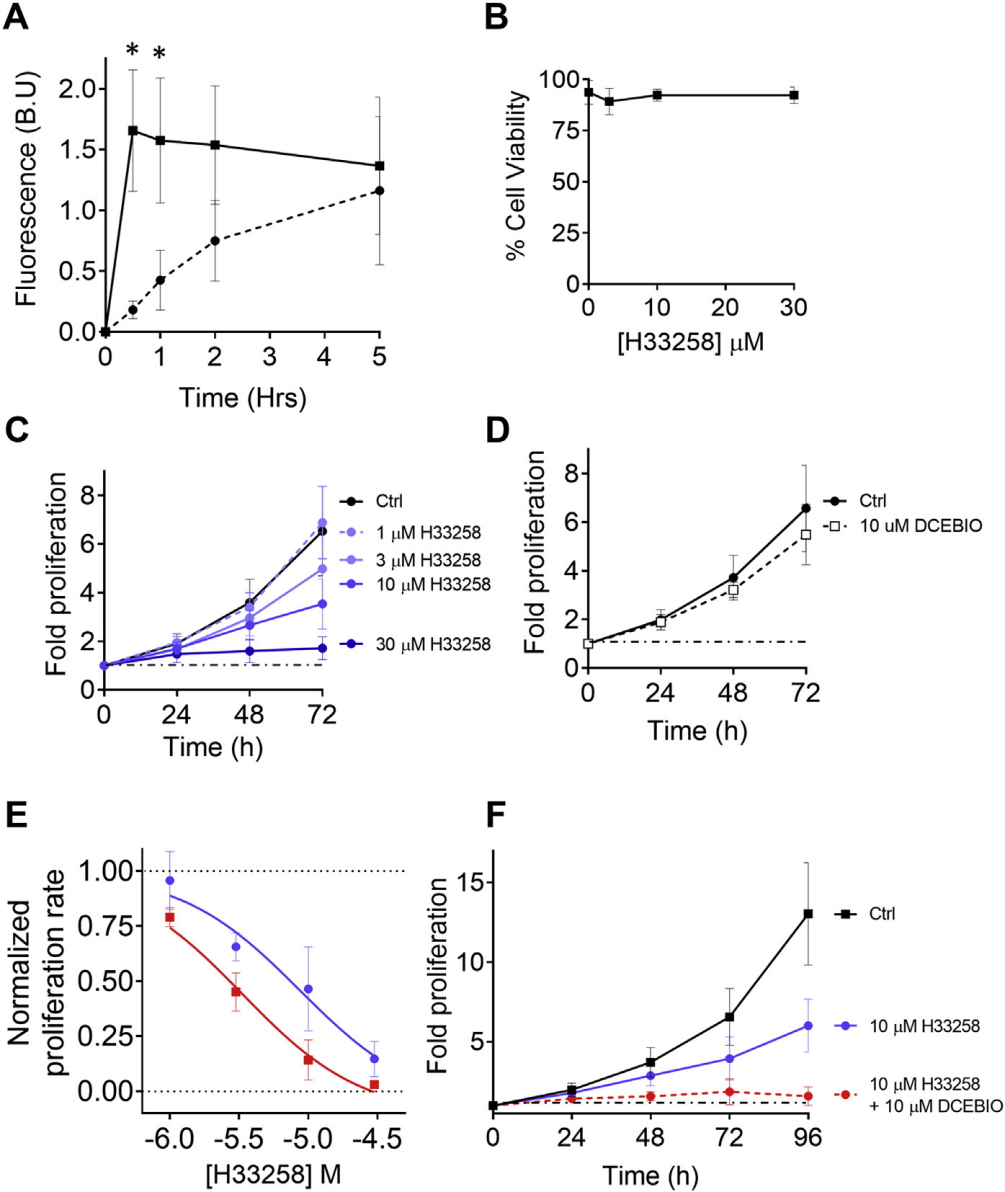
**Long-term effects of K**_**Ca**_**3.1 channel activation on the cytotoxicity of H33258.***A*, cells bathed in culture medium containing H33258 (30 μM) alone (*dashed line*) or H33258 and DCEBIO (10 μM) together (*solid line*) were incubated at 37 °C, 5% CO_2_ over a 5-h period (mean ± SD; n = 3–5). At the indicated time points, H33258 uptake was evaluated by taking the average single cell fluorescence from all cells in three fields of view (normalized to the brightness of at least 5 fluorescent beads, B.U.). Asterisks denotes statistically significant difference ((*p* < 0.0001 and 0.0012 respectively). *B*, pooled data showing negligible effect of increasing concentrations of H33258 on cell viability as assayed by propidium iodide exclusion over a 48 h period mean ± SD; n = 3). *C*, concentration-dependent suppression of CXT2 cell growth by H33258 over the course of a 72-h time period (mean ± SD; n = 3–12). *D*, effect of the K_Ca_3.1 channel activator, DCEBIO (10 μM) on CXT2 cell growth (mean ± SD; n = 3–12). *E*, concentration response curves for H33258-mediated growth inhibition over a 72-h period when applied alone or in combination with DCEBIO (10 μM) mean ± SD; n = 3 to 12. The presence of DCEBIO caused a modest but significant reduction in the IC_50_ of H33258 from 8.7 μM (C.I. 4.5–16.8 μM) to 3.3 μM (C.I. 2.2–5.0 μM) (*p* < 0.0001). *F*, 96-h growth curves of CXT2 cells in the absence and presence of H33258 (10 μM) and DCEBIO (10 μM) (mean ± SD; n = 3–12).

Previously, we observed that while ATP evoked H33258 uptake into cervical cancer cells, ATP did not stimulate measurable uptake and accumulation of the bulkier DNA binding cationic drugs propidium iodide and doxorubicin (1). Consistent with a common mechanism of uptake, SKA 31 also failed to evoke measurable uptake and accumulation of propidium iodide and the slightly smaller cationic DNA binding dye, YO-PRO1 (Fig. 7). SKA 31 did evoke measurable uptake of 4′,6-diamidino-2-phenylindole, but this was surprisingly slower and more modest in rate than for the larger H33258 (Fig. S3, *A*–*B*).

### Effect of sustained K_Ca_3.1 channel activation on cell sensitivity to cytostatic drugs

So far, we have shown that activation of K_Ca_3.1 channels, which are functionally upregulated in cultured cervical cancer cells, facilitate rapid uptake of H33258. This dye is reported to be a DNA-binding topoisomerase inhibitor (32), and so we reasoned that activation of K_Ca_3.1 channels in cells exposed to H33258 might sensitize them to the cytotoxic effects of this drug.

We next compared H33258 uptake in the absence and presence of K_Ca_3.1 activation over a longer time period and, importantly, under conventional cell culture conditions (when in KSFM in a humidified incubator at 37 °C, 5% CO_2_). Cells cultured on 35-mm dishes had H33258 (30 μM) applied directly to the media with either 0.1% dimethyl sulfoxide or the K_Ca_3.1 activator DCEBIO (10 μM). At 30 min, 60 min, 2 h, and 5 h, the dishes were withdrawn from the incubator and H33258 accumulation measured using fluorescence microscopy before being returned to the incubator. Consistent with our experiments conducted in extracellular buffer at room temperature, H33258 uptake occurred significantly more rapidly in the presence of the K_Ca_3.1 activator DCEBIO (10 μM) (Fig. 7*A*) (*p* < 0.0001 and 0.0012 for the 30 min and 60 min time points, respectively). However, after reaching a peak, the response plateaued and eventually converged with the rising passive H33258 accumulation observed in the absence of DCEBIO. At physiological pH of 7.4, a weak base with H33258’s averaged pKa of ∼8 is predicted to be in an uncharged state ∼20% of the time according to the Henderson–Hasselbalch equation, so it is not surprising to see a slow accumulation over long periods in the absence of K_Ca_3.1 activation.

Despite the admittedly narrow window in which H33258 uptake and accumulation was significantly elevated in the presence of K_Ca_3.1 activation, we hypothesized that we might nevertheless see some enhancement of cell sensitivity to the cytotoxic effects of this drug.

Using the propidium iodide exclusion method to assay cell viability, we noted that H33258 caused negligible cell death over the course of 48 h at concentrations up to 30 μM (Fig. 7*B*). In contrast, we saw a clear concentration-dependent suppression of proliferation in cells cultured in H33258 over the course of 72 h (Fig. 7*C*). This is, in fact, consistent with the behavior of canonical DNA-binding anticancer agents, which tend to exert cytostatic rather than cytolytic effects at therapeutically relevant concentrations (see (33) for a discussion of this underappreciated phenomenon).

Having established H33258’s potency as a principally cytostatic agent, we chose a submaximal concentration of 10 μM H33258 and tested whether the inhibition of proliferation evoked could be enhanced in the presence of K_Ca_3.1 channel activation. Cells exposed to DCEBIO (10 μM) alone showed no significant change in proliferation rate over the course of 72 h when compared with cells exposed to vehicle (0.01% dimethyl sulfoxide) (Fig. 7*D*). However, the presence of DCEBIO did cause a modest but significant increase in H33258-mediated growth suppression, as shown by a shift in the concentration response curve of H33258 (Fig. 7*E*). Indeed, whereas a submaximal concentration of H33258 (10 μM) suppressed growth by approximately 50% over a 96-h period, cells co-treated with H33258 and DECBIO (10 μM) showed completely suppressed growth up to 96 h after treatment (Fig. 7*F*).

**Figure 8:**
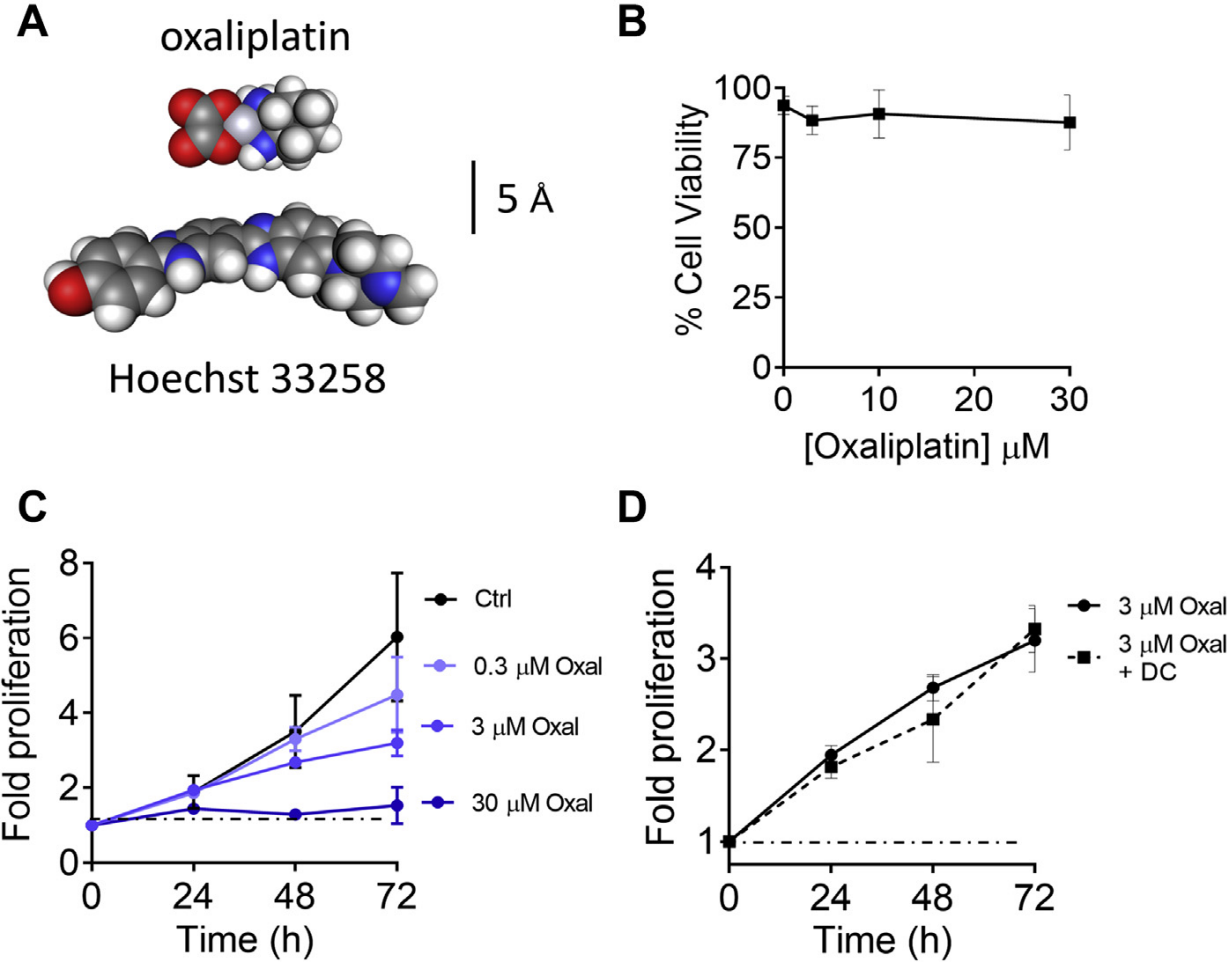
**Lack of effect of K**_**Ca**_**3.1 channel activation on oxaliplatin toxicity.***A*, comparison between the structures of the chemotherapeutic drug, oxaliplatin and H33258. Space-filling models oriented to compare approximate minimum diameters. *B*, the presence of oxaliplatin at clinically relevant concentrations had negligible impact on cell viability, as assayed by propidium iodide exclusion, over a period of 48 h (mean ± SD; n = 6). *C*, sustained exposure to varying concentrations of oxaliplatin over a 72-h period significantly inhibited CXT2 cell proliferation (mean ± SD; n = 3–12). *D*, sustained exposure of CXT2 cells to DCEBIO (10 μM) did not significantly impact the growth suppression caused by co-administration of an IC_50_ concentration of oxaliplatin (3 μM) (mean ± SD; n = 3).

The cisplatin derivative, oxaliplatin, is a common frontline chemotherapeutic. The parent drug is slightly smaller than H33258, and some of its reactive metabolites are cationic (34). We hypothesized that perhaps oxaliplatin or its derivatives would be able to utilize the same K_Ca_3.1-dependent uptake pathway as H33258. Like H33258, and consistent with the activity of many DNA-binding toxins (33), oxaliplatin had little effect on cell viability at therapeutically relevant concentrations (Fig. 8*B*) but potently suppressed cell growth (Fig. 8*C*). However, K_Ca_3.1 channel activation with DCEBIO (10 μM) had no significant impact on the growth suppression caused by a submaximal concentration of oxaliplatin (3 μM) (Fig. 8*D*), providing no evidence that oxaliplatin uptake and accumulation was facilitated by activation of K_Ca_3.1 channels.

## Discussion

In this article, we report evidence that the intermediate conductance Ca^2+^-activated K^+^-ion channel K_Ca_3.1 is functionally upregulated in cervical cancer cells relative to cells isolated from healthy cervical epithelium. Further, we observed that activation of K_Ca_3.1 channels facilitates the rapid uptake and accumulation of a DNA-binding nuclear stain and cytotoxin, H33258, into cervical cancer cells. Moreover, and consistent with our previously published data, this mechanism can be stimulated by activation of G_q_-protein coupled P2Y receptors by extracellularly applied ATP, which causes a PLC-dependent mobilization of Ca^2+^ from the ER and subsequent activation of Ca^2+^-activated K_Ca_3.1 channels. This, in and of itself, is a novel finding within the field of purinergic signaling, where ATP-evoked uptake and accumulation of cationic fluorescent DNA-binding dyes has been widely reported but usually attributed to ATP-gated P2X channel-mediated permeabilization. Members of this channel family, including P2X2 and P2X7, have been consistently reported to conduct species as large as sulfo-rhodamine methanethiosulfonate (4, 5, 6, 35, 36, 37). However, the mechanism of ATP-evoked dye uptake reported here and in our previous article is impermeable to YO-PRO1 and insensitive to P2X7 inhibitors. Further, we found no electrophysiological evidence of P2X7 currents in cervical cancer cells at ATP concentrations up to 3 mM (1). Our data compare well with previous studies suggesting that the reported ATP-evoked permeabilization of macrophages to anionic dyes occurs largely through a P2XR-independent pathway (38, 39). Under our experimental conditions, Ca^2+^ released from the intracellular stores appears to be the main driver of the K_Ca_3.1-dependent H33258 uptake, with little contribution from SOCE. However, this does not rule out the possibility that SOCE could potentially contribute to Ca^2+^-dependent H33258 uptake under certain conditions. The [Ca^2+^]_o_ in our extracellular buffer is matched to the concentration present in the KSFM cell culture media (which is a relatively low 100 μM to attenuate maximize growth and prevent terminal differentiation). Under these conditions, SOCE makes a limited contribution to the ATP-evoked [Ca^2+^]_i_ elevation (Fig. S1, *D*–*E*). Investigating the role of SOCE in the ATP-evoked H33258 uptake at higher [Ca^2+^]_i_ is complicated by a concentration-dependent suppression of ATP-evoked H33258 uptake by [Ca^2+^]_o_ [1]. The reason for this is unclear but appears to be specific to the ATP-evoked uptake, as direct activation of K_Ca_3.1 with SKA 31 still evoked substantial H33258 uptake in physiological [Ca^2+^]_o_ (Fig. S2*C*).

The most direct explanation for the K_Ca_3.1 dependence of H33258 uptake is that the channel itself conducts entry of this cationic dye. The fact that transient expression of the K_Ca_3.1 gene in HEK293 cells was alone able to confer SKA 31-evoked H33258 uptake in the cells would seem consistent with this hypothesis. However, the ability for K^+^ selective channels to effectively discriminate between the small monoatomic cations, principally K^+^ and Na^+^, questions the plausibility of large cations penetrating the pore of these particular proteins. Nevertheless, it should be noted that the protracted time course of our experiments is such that it would likely require only a very small relative conductance of H33258 to account for the slow accumulation of the dye observed.

It is unlikely that H33258 uptake is because of a signaling event downstream of K_Ca_3.1-dependent membrane hyperpolarization, as the uptake was not reproduced by direct hyperpolarization induced by the K^+^ ionophore valinomycin. However, we did observe a complete inhibition of H33258 uptake in the presence of K_Ca_3.1 channel activation when the membrane potential was clamped near zero by substituting extracellular NaCl with KCl. This could be because of the need for a negative electromotive force to pull the cationic H33258 into the cell or increased competition for the permeating pathway by the presence of high extracellular (as well as intracellular) K^+^.

Given the ongoing controversy surrounding the question of how large cationic dyes traverse the membrane during the activation of other ion channels (see (40, 41)), we are reluctant to state unequivocally that H33258 permeates the pore of K_Ca_3.1 channels on the strength of the evidence presented here. It is unlikely that a mutation in the gene for K_Ca_3.1, KCNN4, in cancer cells is giving rise to a H33258 permeable mutant in cancer cells, as our data show that the wild-type human K_Ca_3.1 transfected into HEK293 cells still facilitates H33258 uptake. Nevertheless, future biophysical studies beyond the scope of the current article are needed to assess the potential interactions between H33258 and the K_Ca_3.1 channel pore itself, as has been done for the interactions of the lidocaine derivative QX-314 and the TRPV1 channel (42).

In keeping with our previous results showing that the ATP-evoked uptake of H33258 was largely confined to cancerous populations of cervical epithelial cells (1), we show here that K_Ca_3.1-dependent H33258 uptake is also primarily observed in cervical cells obtained and cultured from cancerous tissue. That K_Ca_3.1 appears to be functionally upregulated in cervical cancer cells is further supported by our data showing a substantially increased occurrence of SKA 31 and TRAM 34-sensitive K^+^ currents and K^+^-mediated hyperpolarization responses in cancer cells relative to cervical epithelial cells obtained from the ectocervix and TZ of healthy tissue. K_Ca_3.1 is known to regulate cell migration, cell growth, and secretion and have been implicated in a number of cancers (9, 10, 11, 12, 13, 14, 15). While we saw no significant effect of the K_Ca_3.1 activator, DCEBIO, on cell growth in our study, future studies are necessary to thoroughly examine the true extent of K_Ca_3.1 channel upregulation in cervical cancer cells and whether this confers advantages in terms of cell growth, migration, and survival. Indeed, our reliance on pharmacological tools probing the functional consequence of K_Ca_3.1 activity in cervical epithelial cells is certainly a limitation of this study. In addition to possible off-target effects of the drugs used, our work does not distinguish whether the observed functional increase in K_Ca_3.1 activity is because of increased protein expression or perhaps changes in membrane trafficking. Future work aims to more closely investigate K_Ca_3.1 channel expression in healthy and diseased cervical epithelial cells, with an emphasis on examining the effects of RNA knockdown and KCNN4 gene deletion on the growth, migration and survival of healthy and cancerous cervical epithelial cells, as well as their capacity to uptake small cationic toxins like H33258.

Having established that activation of K_Ca_3.1 channels caused marked uptake and accumulation of a known DNA-binding cytotoxin, we hypothesized that K_Ca_3.1 activators might facilitate selective sensitivity of cancer cells to small DNA-binding drugs. In this, our rationale was not that different from a previous series of studies successfully using the cation nonselective channel, TRPV1, to facilitate the delivery of a local anesthetic, QX-314, into nociceptor neurons, thereby selectively suppressing pain and itch respectively (42, 43, 44). Consistent with reports for the majority of DNA-binding anticancer drugs (33), we found that both H33258 and the cisplatin derivative, oxaliplatin, chiefly inhibit cell growth, causing negligible cell death at micromolar concentrations over the course of 72 h. The presence of K_Ca_3.1 activators had no effect on oxaliplatin-mediated growth suppression, providing no evidence that cellular uptake of either the parent drug or its metabolites was enhanced by this mechanism. In contrast, we observed a modest but significant sensitizing effect of K_Ca_3.1 activation on H33258-mediated growth suppression.

Based on our short time point experiments, we were perhaps optimistic on the likely impact of K_Ca_3.1 activation on the long-term effects of H33258 on cell growth. A confounding factor in these experiments was the fact that, while little H33258 uptake was observed in cells over the course of an hour, we did observe significant nuclear accumulation over the course of several hours, regardless of whether K_Ca_3.1 activators were present. Indeed, within 5 h, there was no significant difference in the H33258 nuclear staining intensity between cells treated with H33258 alone *versus* those treated with both K_Ca_3.1 activators and H33258. The period in which K_Ca_3.1 activation elevated H33258 accumulation constitutes a relatively short period, likely explaining the modest impact on cell sensitivity to H33258. Nevertheless, cationic cytotoxins with a much lower passive permeability might more efficiently be delivered through this K_Ca_3.1-dependent mechanism.

In light of the data presented here and previous studies supporting the potential for drug delivery through stimulated endogenous transmembrane transport mechanisms, the possibility that K_Ca_3.1 channel activation might provide an additional means of sensitizing cancer cells to cytotoxins is one worth exploring further. This is particularly so given that these channels have been reported to be functionally upregulated in several cancers. Relevant to cervical cancer, The Cancer Genome Atlas reports that K_Ca_3.1 mRNA levels are elevated 5-fold in cervical cancer specimens (n = 306) relative to normal cervix (n = 3), which is consistent with a trend shown in our own preliminary qPCR experiments (Fig. S3*C*). From The Cancer Genome Atlas database, other cancers that appear to significantly overexpress K_Ca_3.1 mRNA relative to healthy tissue include cholangiocarcinoma (4.2-fold), kidney renal clear cell carcinoma (4-fold), lung adenocarcinoma 9.3-fold), pancreatic adenocarcinoma (6.1-fold), and, strongest of all, thyroid carcinoma (27-fold). The fact that K_Ca_3.1 upregulation is not an uncommon occurrence in various cancers of course raises the question of whether the presence of this channel presents some type of tumorigenic advantage. Inhibition of the K_Ca_3.1 channels has been reported to suppress proliferation of prostate cancer cells (45, 46, 47) and to suppress migration in glioblastoma cells (9, 14, 48, 49). Future work in our laboratory aims to investigate more thoroughly the incidence and influence of upregulated K_Ca_3.1 channels in the context of cervical cancer to determine if, and to what degree, this protein might constitute a therapeutic target in its own right.

## Experimental procedures

### Cell culture and transfection

Cervical carcinoma cell lines (CXT) and nonmalignant cervical epithelial cell strains (HCX) were derived from tissues purchased from the Cooperative Human Tissue Network as described elsewhere (50). The samples were surgically removed for other purposes, and patient origin was not identified. Both cancer and nonmalignant cells were cultured in KSFM supplemented with 50 μg/ml streptomycin, 50 units/ml penicillin G, 20 μg/ml gentamicin, 5 ng/ml human recombinant epidermal growth factor, and 50 μg/ml bovine pituitary extract. Cells from nonmalignant cervix were isolated from both the ectocervical region and the TZ, where most cervical cancer originates, and used within passages 1 to 3. HEK293 cells were cultured in Dulbecco’s modified Eagle’s medium supplemented with 10% fetal bovine serum, 2 mM L-Glutamine, 50 units/ml penicillin G, and 50 μg/ml streptomycin. All cells were maintained in a humidified atmosphere with 5% CO_2_ at 37 °C. For imaging and patch clamp experiments, cells were seeded onto 13 mm borosilicate glass coverslips and allowed to attach for at least 24 h.

HEK293 cells were co-transfected with plasmids containing K_Ca_3.1 and green fluorescence protein cDNA using Effectene according to the manufacturer’s instructions (Qiagen). Cells were plated onto 13 mm borosilicate glass coverslips (Gold Seal, Becton-Dickinson) 24 h posttransfection. Further experiments were conducted about 48 h post transfection.

### Fluorescence imaging

Glass coverslips seeded with cells were placed onto a perfusion bath on the stage of Nikon TE200 epifluorescence microscope. Unless otherwise specified, cells were perfused with an extracellular solution of composition (mM): 140 NaCl, 5 KCl, 10 HEPES, 10 glucose, 0.1 CaCl_2_ (pH 7.4 with NaOH). Drugs were added in final concentration through peristaltic pump-mediated perfusion. Equimolar KCl was substituted for NaCl in the above solution to clamp the resting membrane potential to ∼0 mV.

Images were acquired using Hamamatsu ORCA-ER-1394 camera. Fluorescence over time was recorded using appropriate filters for the fluorophore and analyzed as the mean from all the cells in a field of view using μManager (Professor R. Vale laboratory, University of California, San Francisco, CA).

For assessing dye uptake, images were taken at 0.1 Hz with 200 ms exposure through light attenuating neutral density filters. Under these conditions, we observed no signs of phototoxicity during the course of our experiment, even when ultraviolet was used for excitation. The dyes H33258 and 4′,6-diamidino-2-phenylindole were visualized through a 350 nm excitation/450 nm emission filter.

For recording changes in membrane potential with the voltage-sensitive dye, DiSBAC_2_(3), cells were incubated at room temperature for 20 min in extracellular solution containing DiSBAC_2_(3) (100 nM) before imaging. Imaging experiments were then conducted in the continued presence of DiSBAC_2_(3) (100 nM). Changes in membrane potential were measured as the relative change in fluorescence (fold over basal, F/F_0_) from the resting level. Fluorescence was observed through a 488 nm excitation/510 nm emission filter.

To monitor H33258 uptake and accumulation over longer time courses, we controlled for variations in the mercury light source by including Carboxy Bright Blue microspheres (Polysciences) in the culture medium (see (51)). The mean H33258 fluorescence intensity averaged across all cells in the field of view was then normalized to the fluorescence intensity of at least five beads measured in the same dish using the same ultraviolet filter set. Fluorescence amplitude was then reported in bead units.

### Patch clamp electrophysiology

Cells on glass coverslips were placed in a bath mounted on the stage of stage of an Olympus IX-51 inverted microscope. Cells were perfused throughout with an extracellular buffer of composition (mM): 140 NaCl, 5 KCl, 10 HEPES, 10 glucose, 1.8 CaCl_2_, and 1 MgCl_2_ (pH 7.4 with 4 mM NaOH). Whole cell broken patch electrophysiology was performed on single cells using 3 to 5 MΩ resistance glass pipettes filled with the appropriate intracellular solution. Drugs were applied by gravity flow *via* a Perfusion Fast-Step System SF-77 (Warner Instruments). To assess TRAM 34 inhibition of Ca^2+^-activated currents, cells were patched and held at −40 mV membrane potential using pipettes filled with an intracellular pipette solution in which the free Ca^2+^ concentration was predicted to be ∼600 nM using MaxChelator (52). This solution was of composition (mM): 123 K^+^-Aspartate, 15 KCl, 10 HEPES, 10 EGTA and 9 CaCl_2_ (pH 7.3 with 38 mM KOH). Under these conditions, immediate large outward currents were observed upon breaking into the whole cell configuration. The reversal potential of the outward current was determined with a voltage ramp protocol from −140 mV to 60 mV. To assay for currents evoked by the K_Ca_3.1 agonist SKA 31, cells were patched and held at −40 mV membrane potential using pipettes filled with an intracellular pipette solution in which the approximate free Ca^2+^ concentration was <100 nM. This solution was of composition (mM): 140 KCl, 10 HEPES, 0.05 EGTA (pH 7.3 with 4 mM KOH). Data were recorded using AxoGraphX (Axograph, Foster City, CA) software and analyzed offline using IgorPro (Wavemetrics, Inc, Lake Oswego, OR).

### Cell viability assay

Cells seeded onto 35-mm culture dishes received vehicle or drug treatment directly to their culture medium and were then incubated for 48 h (5% CO_2_, 37 °C). The media was replaced, and propidium iodide (10 μM) added to test for viability as a function of dye exclusion. After a 10-min incubation at room temperature, cell viability within each dish was assessed based on the number of propidium iodide–positive cells manually counted across three fields of view under the x10 microscope objective attached to a Nikon TE200 epifluorescence microscope (fluorescence observed through a 530 nm excitation/620 nm emission filter). Counts were made by an investigator blinded to the treatment regimen.

### Cell proliferation

Cells seeded at low density onto 35-mm dishes (∼100 cells/cm^2^) received vehicle or drug treatment directly to their culture medium. For each dish, cells from three marked fields of view were immediately counted under a x10 objective by an investigator blinded to the treatment regimen. Every subsequent 24 h, the investigator counted the cells in the same three fields of view over a 3- to 4-day period. Cell growth was then assessed as the mean fold increase in cell number obtained from duplicate experiments.

### Reagents

9-Phenanthrol, ATP, oxaliplatin, thapsigargin, and valinomycin were purchased from Sigma-Aldrich (St Louis, MO). DiSBAC_2_(3) was purchased from ThermoFisher (Waltham, MA). A-967079, 1,2-bis(2-aminophenoxy)ethane-N,N,N′,N′-tetraacetic acid tetrakis(acetoxymethyl ester), DCEBIO, SKA 31, TRAM 34, triphenylphosphine oxide, and U73122 were purchased from Tocris Bioscience (Bristol, United Kindgom).

### Analysis and statistics

For all histograms, individual data points are shown with the mean and standard deviation of the sample (mean ± SD). Dye uptake traces are shown as the single mean average with SD bars. GraphPad Prism (GraphPad Software, Inc, La Jolla, CA) was used to produce graphs and perform statistical analysis. Peak dye uptake rates (*d/dt*) were compared by one-way ANOVA with Tukey’s multiple comparisons test. Fractional dye uptake response percentages between cells were compared using either an unpaired two-tailed Student’s *t*-test or one-way ANOVA depending on number of samples being compared. In all cases, our threshold for statistical significance was *p* < 0.05, and individual *p* values are reported in the results text and/or legends where appropriate. Two-way ANOVA was used to compare concentration response curves for H33258-mediated inhibition of cell growth in the absence and presence of the K_Ca_3.1 channel activator, DCEBIO. Half-maximal inhibitory concentrations of H33258 (IC_50_) are reported with 95% confidence intervals. The *p* values and goodness-of-fit (R^2^) are indicated in the relevant Figure legends.

## Data availability

All relevant data are presented in the main paper and supporting information of this manuscript.

## Conflict of interest

The authors declare that they have no conflicts of interest with the contents of this article.
