# Supplementary material for: KCa3.1-dependent uptake of the cytotoxic DNA-binding dye Hoechst 33258 into cancerous but not healthy cervical cells
Source: J Biol Chem. 2020 Nov 23;296:100084. doi: 10.1074/jbc.RA120.013997 (PMC7948979; doi:10.1074/jbc.RA120.013997)
Supplement: Figures S1–S3 [file mmc1.pdf]

SUPPORTING INFORMATION FIGURE 1

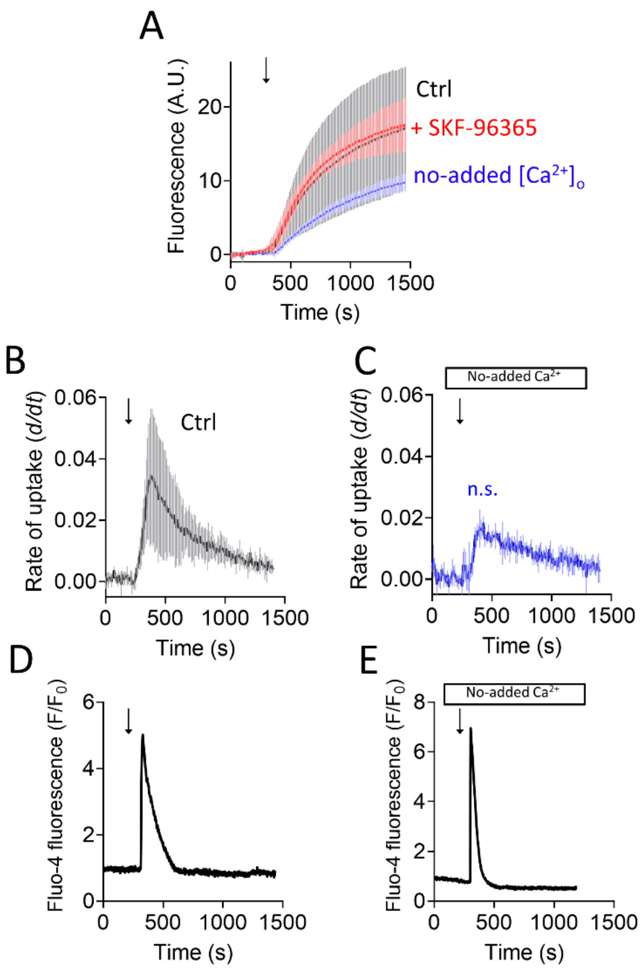

Supporting information Fig. 1. A) Pooled average fluorescence intensity data showing level of ATP-evoked H33258 uptake and nuclear accumulation in cells over time under control conditions (black, n = 6), in the absence of added extracellular Ca^{2+} (blue, n = 3) and in the presence of the store-operated Ca^{2+} channel (SOC) inhibitor SKF-96365 (red, 10 µM; n = 4). ATP addition indicated by black arrows. B and C) These traces show the ATP-evoked H33258 uptake rates obtained in the presence (B) and absence (C) of added extracellular Ca^{2+} (duration indicated by the horizontal bar) which are presented for comparison with the time-lapse Ca^{2+} imaging traces obtained in D and E in the same extracellular solutions. The asbence of extracellular Ca^{2+} caused a modest but not statistically significant decline in the peak rate of ATP-evoked H33258 uptake (p=0.21). D and E) Cells were loaded with 2 µM Fluo-4/AM (Thermo Fisher Scientific, Waltham MA) for 30 minutes, then washed with extracellular buffer and allowed to undergo de-esterification at 37^0C for a further 15 min. Cell fluorescence was imaged at 0.5 frames/second using a FITC filter and a x20 objective. In D, cells were bathed in extracellular buffer containing 100 µM added Ca^{2+} (similar to the concentration present in KSFM culture medium) and stimulated with 100 µM ATP. In E, cells were bathed in an extracellular solution without added Ca^{2+} for the period depicted by the horizontal bar.

SUPPORTING INFORMATION FIGURE 2

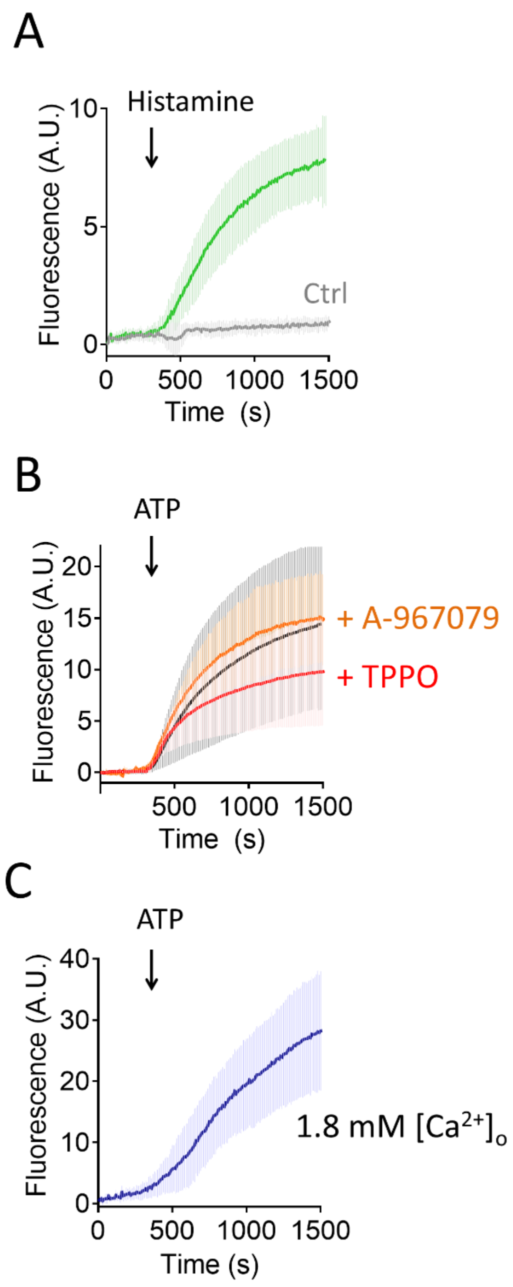

Supporting information Fig. 2. A) Pooled average fluorescence intensity data showing that activation of endogenous G_q protein-coupled epithelial H_1 receptors in CXT2 cervical cancer cells stimulated H33258 uptake (mean ± s.d; n = 4). B) ATP-evoked H33258 uptake in CXT2 cells was not significantly suppressed by drugs inhibiting Ca^{2+}-activated TRPA1 (1 μM A967079), or TRPM5 (100 μM Triphenylphosphine oxide). C) SKA 31-evoked H33258 uptake in CXT2 cells in the presence of extracellular solution containing 1.8 mM extracellular Ca^{2+}.

SUPPORTING INFORMATION FIGURE 3

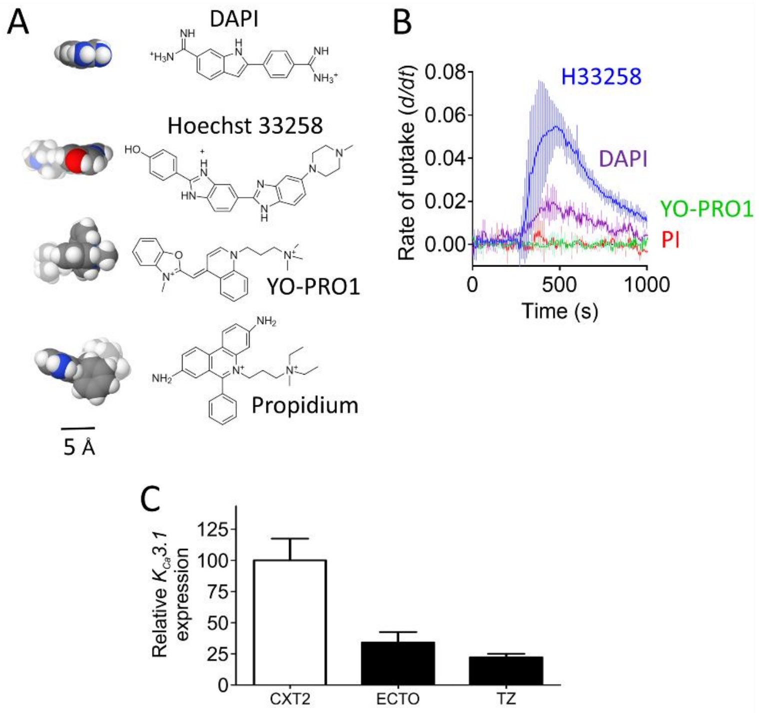

Supporting information Fig. 3. A) Comparison of structures of different fluorescent DNA-binding cations. Chemical structures are shown right with the energy minimized minimum diameter perspectives shown left. The white scale bar represents 50 µm. B) Pooled data comparing the rates of SKA 31-evoked uptake of the four dyes shown in (A). C) Preliminary results from one of two qPCR experiments conducted on ECTO, TZ, and cancerous CXT2 cell culture samples (N=2, mean and s.d. of pooled replicates shown). Briefly, cells were lysed using 1 ml of TRIzol Reagent (Ambion), homogenized by pipetting and transferred to a microcentrifuge tube. RNA was extracted and precipitated from the Trizol reagent following the manufacturers protocol. After precipitation, RNA was washed twice in 75% ethanol and allowed to air dry before being solubilized in 60 µl of nuclease-free water (Ambion). Concentration and purity of RNA samples obtained were determined by measuring the A260/280 values using a Spectramax I3x microplate reader with Spectradrop micro-volume microplates (Molecular Devices). The RNA samples were treated with RNase free DNAse I (New England BioLabs; 1 unit of DNAse I per 5 µg of RNA) for 20 min at 37 °C to minimize genomic DNA contamination. Subsequently, DNAse was inactivated by heating at 75 °C for 10 minutes in presence of 5 mM EDTA (Sigma-Aldrich). To create cDNA library from mRNA, reverse transcription was performed using the High-Capacity cDNA Reverse Transcription Kit (Applied Biosystems) following the manufacturer's protocol. PCR amplification was performed using Forget-Me-Not EvaGreen qPCR Master Mix (Biotium) and a CFX96 Touch Real-Time PCR Detection System (Biorad). The PCR reactions contained 50 ng cDNA, 5 µM of forward and reverse primer, and 5 µl of 2X master mix in 10 µL of reaction volume. The primers used in this work are as follows: $KCa_{3.1}$ (forward, 5'-AACTGGCATCGGACTCATGGTTCT-3'; reverse, 5'-AGTCATGAACAGCTGGACCTCCTT-3') (52); GAPDH (forward, 5'- TCAACGACCACTTTGTCAAGC-3'; reverse, 5'-TACTTTATTGATGGTACATGACAAGG-3') (53). The PCR conditions include cDNA denaturation for 5 min at 95 °C, followed by 40 cycles of 10 s at 95 °C and 45 s at 60 °C. After completion of 40 cycles a melting curve analysis was performed to ensure the specificity of amplified products. Relative $KCa_{3.1}$ gene expression was estimated by the comparative Ct method using GAPDH or actin genes as control.
